# Acid–Base Status in Critically Ill Patients: Physicochemical vs. Traditional Approach

**DOI:** 10.3390/jcm14093227

**Published:** 2025-05-06

**Authors:** Arianna Ciabattoni, Davide Chiumello, Simone Mancusi, Tommaso Pozzi, Alessandro Monte, Cosmo Rocco, Silvia Coppola

**Affiliations:** 1 Department of Anesthesia and Intensive Care, ASST Santi Paolo e Carlo, San Paolo University Hospital, 20142 Milan, Italy; 2 Department of Health Sciences, University of Milan, 20122 Milan, Italy

**Keywords:** acid–base, critical illness, physicochemical approach, standard base excess, intensive care unit

## Abstract

**Background/Objectives**: Critically ill patients can often present acid–base alterations. The aim of this study was to evaluate the prevalence and the time-course of acid–base alterations on intensive care unit (ICU) admission and on day one by the traditional standard base excess (SBE)-based and the Stewart methods in mechanically ventilated patients. **Methods**: A prospective observational study enrolling mechanically ventilated patients in the ICU was conducted. Arterial blood gas analysis, blood and urine samples were obtained on ICU admission and on day one. Plasmatic and urinary acid–base variables were compared among acidemic, alkalemic and patients with normal pH. The agreement between the SBE-based and Stewart methods was assessed at ICU admission and on day one. **Results**: One hundred and seventy-two patients were enrolled. On ICU admission, 55 (32%), 29 (17%) and 88 (51%) patients had acidemia, alkalemia and a normal pH, respectively. On day one, 12 (7%), 48 (28%) and 112 (65%) patients had acidemia, alkalemia and a normal pH with lower values of paCO2 and albumin. According to the SBE and Stewart approaches, the occurrence of metabolic acidosis was similar (24% vs. 35%), as well as the rate of metabolic alkalosis (16% vs. 23%) on ICU admission; on day one, the occurrence of metabolic acidosis was different (12% vs. 35%), as well as the rate of metabolic alkalosis (35% vs. 14%). The agreement between methods was estimated to be low both on ICU admission and on day one. **Conclusions**: Up to 50 % of mechanically ventilated patients presented acid–base derangements, mainly due to acidemia on ICU admission and to alkalemia after 24 h, secondary to alterations in carbon dioxide and plasma albumin. The agreement between the traditional and Stewart approaches was poor. The Stewart approach could be more accurate in detecting the acid–base disturbances in critically ill patients characterized by changes of mechanical ventilation and fluid administration.

## 1. Introduction

Critically ill patients can present acid–base alterations mainly due to the presence of hemodynamic instability, massive alteration in intravascular volume, crystalloid infusion, acute respiratory failure, hypoperfusion, hepatic dysfunction and kidney failure [1]. At intensive care unit (ICU) admission, the most frequent acid–base alterations are the metabolic acidosis and metabolic alkalosis [1,2,3,4,5,6]. The changes in blood pH in both conditions are associated with a higher mortality [7]. The degree of pH alterations and the rate of correction/resolution were associated to the hospital outcome [8].

The regulation of the acid–base equilibrium is fundamental in order to maintain a normal cell function and metabolism [4,9]. The renal and respiratory system actively participate to maintain the acid–base homeostasis [9].

The traditional approach for the assessment of acid–base equilibrium is based on the Henderson–Hasselbalch equation in which the carbon dioxide (PCO_2_) and the concentration of actual bicarbonate (HCO_3_) are the determinants of the pH [10]. Thus, the alterations in acid–base equilibrium are caused by an increase or decrease in PCO_2_ and/or in HCO_3_. Criticism of this approach mainly includes the absence of independence of actual bicarbonate from the respiratory component and failure of the quantification of buffer other than bicarbonate [11]. Therefore, to eliminate the respiratory component, the standard bicarbonate was first introduced, representing the plasma bicarbonate concentration after equilibration at a PCO_2_ of 40 mmHg. Then, to take into account the buffer effect of weak non-carbonic acids, i.e., proteins which normally contribute to buffering with 14–16 negative charges per liter, Singer and Hastings introduced the buffer base (BB), which is the sum of all buffer anions. Unfortunately, there is a physiologic inter-subject variability due to different non-carbonic buffer concentrations; so, to overcome this limitation, Siggaard-Andersen introduced the base excess (BE), i.e., the “excess” (either positive or negative) of the actual BB as compared to the normal BB. BE is the amount of acid/base (mmol/L) that must be added to the blood sample to reach a pH of 7.40 in standardized conditions (PCO_2_ 40 mmHg, 37 °C) [12].

In most cases, this so-called traditional approach which includes the determination of the standard base excess (SBE), bicarbonate concentration in the plasma (HCO_3_^−^) and pCO_2_ is accurate; however, when electrolytes or protein abnormalities are present, it might be clinically misleading [13]. An alternative mathematical model based on physicochemical principles described by Stewart has been developed. This Stewart method proposes that the acid–base equilibrium is determined by three independent variables: the strong ion difference (SID), the PCO_2_ and the weak acids [14,15]. Compared to the traditional approach, the pH (i.e., hydrogen ion concentration) and bicarbonate ions are not independent variables but are determined by other factors. According to this model, a whole evaluation of the acid–base status should require an arterial blood gas analysis, serum electrolyte and albumin measurement.

When Stewart’s method was compared to the traditional, it allowed one to detect and to quantify the most complex acid–base disturbances in critically ill patients, with also a higher correlation with the outcome, but without offering any clear diagnostic benefit [6,16,17,18].

The aim of this prospective study was to evaluate in mechanically ventilated critically ill patients the occurrence of acid–base alterations (plasma and urine) on intensive admission and their variations after the first 24 h of intensive care unit (ICU) stay according to the traditional approach and Stewart’s method. The secondary aim was to investigate the role of kidney function on the occurrence and the progression of acid–base alterations.

## 2. Materials and Methods

### 2.1. Study Population

This was a prospective, observational, single-center cohort study of critically ill patients admitted to the ICU of San Paolo University Hospital from June 2023 to June 2024. This study was approved by the local authorities (2023/ST/032). All sedated and mechanically ventilated adult patients admitted to the ICU for acute medical or surgical conditions or for post-operative care after elective surgery were considered eligible for the study and consecutively enrolled. Exclusion criteria were pregnancy, patients who received diuretic or steorid therapy after ICU admission, the need for renal replacement therapy and life expectancy less than 48 h.

### 2.2. Study Protocol

On ICU admission, patients were maintained deeply sedated and mechanically ventilated; if admitted for post-operative monitoring, patients were ventilated using the same intraoperative ventilatory setting, with the respiratory rate (RR) adjusted to maintain an end-tidal carbon dioxide partial pressure (EtCO_2_) between 35 and 40 mmHg. Patients requiring care for acute medical or surgical conditions were ventilated according to clinical judgement.

### 2.3. Data Collection

On ICU admission, anthropometric and demographic data, SOFA [19] and APACHE III [20] scores were collected as well as the ICU admission diagnosis, clinical history and chronic pharmacological therapy of each patient. Thirty minutes after admission to the ICU, an arterial blood gas analysis was performed; blood and extemporary urine samples were also sent for biochemical and electrolyte analysis. The same set of measurements was performed the day after ICU admission at a pre-determined time in the morning (day one). Blood gas, blood and urine sample analyses were performed using the same analyzers.

Urine output was calculated considering the previous 3 h before the ICU admission.

Further details of the measurements are reported in the Supplementary Material.

### 2.4. Calculate Variables

Bicarbonate concentration was calculated according to the Henderson–Hasselbalch equation [21]. Standard base excess (SBE) was calculated according to Zander’s formula [22].

Apparent strong ion difference ([SID]a) was calculated as follows:

$$\left[SID]a=\left[{Na}^{+}\right]+\left[K^{+}\right]+2\times \left[{Ca}^{++}\right]+2\times \left[{Mg}^{++}\right]-\left[{Cl}^{-}\right]-[{Lac}^{-}\right]$$

where [Na^+^], [K^+^], [Ca^++^], [Mg^++^], [Cl^−^] and [Lac^−^] are the plasma concentrations of sodium, potassium, calcium, magnesium, chloride and lactates, respectively, in mEq/L [14].

Effective strong ion difference ([SID]e) was calculated as follows:

$$\left[SID\right]e=\left[HCO_{3}^{-}\right]+\left[Alb\right]\times \left(0.123\times pH-0.631\right)+[PO_{4}]\times (0.309\times pH-0.469)$$

where [Alb] is plasma albumin concentration in g/L and [PO_4_] is plasma phosphate concentration in mMol/L [23].

The strong ion gap ([SIG]) was calculated as the difference between the apparent and the effective [SID].

Urinary [SID] ([SID]u) was estimated as follows:

$$\left[SID\right]u=\left[{Na}^{+}\right]u+\left[K^{+}\right]u-\left[{Cl}^{-}\right]u$$

where [Na^+^]u, [uK^+^]u and [uCl^−^]u are the urinary concentrations of sodium, potassium and chloride, respectively, in mEq/L.

Glomerular filtration rate was estimated according to Levey et al. [24].

### 2.5. Patient Stratification

To investigate the difference among patients with acidemia, alkalemia or no pH abnormalities, they were stratified by pH into low (pH < 7.35), normal (7.35 ≤ pH ≤ 7.45) or high pH (pH > 7.45), both at admission and after 24 h.

To investigate the relative performance of the SBE-based and Stewart methods in identifying metabolic acidosis and alkalosis, patients were stratified by SBE into low (SBE < −3.0), normal (−3.0 ≤ SBE ≤ 3.0) and high SBE (SBE > 3.0) groups and by apparent SID into low (SIDa < 38), normal (38 ≤ SIDa ≤ 42) and high SID (SIDa > 42) groups, both at admission and after 24 h (Figure S1).

To investigate the role of renal function on acid–base disturbances, patients were stratified according to their estimated glomerular filtration rate into those with (eGFR < 90mL/min) or without previous renal impairment based on the first available creatinine value (the first measured creatinine at the emergency department for acute critically ill patients or the preoperative creatinine for elective surgical patients).

### 2.6. Statistical Analysis

Continuous data are expressed as mean ± standard deviation or median [interquartile range], as appropriate (Shapiro–Wilk’s test); categorical data are expressed as percentage (number). Differences in continuous variables between ICU admission day and the day after were assessed by paired Student’s T test for repeated measures or paired Wilcoxon–Mann–Whitney U test, as appropriate. Differences between patients with low (pH < 7.35), normal (7.35 ≤ pH ≤ 7.45) or high pH (pH > 7.45) were assessed by one-way ANalysis Of VAriance (ANOVA) or Kruskal–Wallis test, as appropriate. Differences between patients with or without prior renal function impairment were assessed by Student’s T test or Wilcoxon–Mann–Whitney U test, as appropriate. The rates of patients with low, normal or high SBE or apparent SID were compared by χ^2^ test. The agreement between SBE and SIDa in identifying patients with metabolic acidosis, metabolic alkalosis or no metabolic acid–base derangement was assessed by Fleiss’κ. The agreement between numerical values of SBE and SIDa was evaluated by Bland–Altman plot and Lin’s Concordance Correlation Coefficient (LCCC); however, because SIDa and SBE are not numerically comparable, as SBE should be a surrogate of the difference between normal SIDa and actual SIDa, a normal SID of 42 mEq/L was assumed to computed the Δnormal–actualSIDa as normal SIDa–actual SIDa.

To investigate the role of possible confounders on the numerical association between SBE and aSID, we used a univariate and then a multivariate linear model evaluating the association between changes in SBE and aSID from ICU admission and day one (ΔSBE and ΔSIDa, respectively) adjusting for relevant covariates (changes in albumin concentration, changes in carbon dioxide partial pressure, total amount of administered fluid, changes in creatinine).

To investigate the role of possible confounders on the diagnostic performance of SBE and aSID, we used a univariate logistic regression model to determine sensitivity, specificity and the area under the curve (AUC) of each variable in determining pH, categorized as acidemic (pH < 7.38) or alkalemic (pH > 7.42), in patients without a primary respiratory acid–base disorder, defined either as the combination of a pH > 7.44 with a PaCO_2_ < 36 mmHg or a pH < 7.36 with a PaCO2 > 44 mmHg, which were temporarily excluded from the dataset. We subsequently used the same approach in a multivariate logistic regression, adjusting for relevant covariates (albumin concentration, carbon dioxide partial pressure, total amount of administered fluid, creatinine), for both SBE and SIDa.

A *p* value less than 0.05 was considered as statistically significant.

## 3. Results

### 3.1. On ICU Admission

One hundred and seventy-two patients were prospectively enrolled and their main clinical characteristics are shown in Table 1.

Of these, 121 (70%) were admitted electively from the operating room after surgery, while 22 (13%) and 29 (17%) were admitted for sepsis and acute respiratory failure, respectively. Median APACHE III and SOFA scores were 9 [7–13] and 3 [1–6], respectively. The all-cause hospital mortality rate was 7%. On ICU admission, the median arterial pH, PaCO_2_, HCO3^−^ and SBE were 7.38 [7.33–7.43], 42 [37–46] mmHg, 24.5 [22.6–26.6] mMol/L and −0.6 [−3.0–1.7] mMol/L, respectively (Table 2).

Apparent and effective SIDs, according to Stewart’s approach, were 39.2 [37.5–41.8] and 35.7 [33.3–38.0] mEq/L, respectively. The urine output was 0.7 mL/kg/h with a pH of 6.5 and a urinary SID of 58 mEq/L (Table S1).

Dividing the population according to the arterial pH, 55 (32%), 29 (17%) and 88 (51%) patients had acidemia, alkalemia and a normal pH, respectively (Table 3). Acidemic patients had a significantly higher PaCO_2_ and lower SBE with similar SIDa but higher SIG. Plasma albumin was significantly lower in the alkalemic patients. Urinary output and SIDa were similar among pH groups while only urine chloride was significantly lower in the alkalemic patients (Table 3).

On ICU admission, the occurrence of metabolic acidosis according to the SBE and Stewart approaches (24% vs. 35%, respectively) was similar; similarly, the rate of metabolic alkalosis was not different (16% vs. 23%, respectively) (Table 4). However, the agreement between methods was insufficient (κ = 0.08); indeed, among 43 patients with reduced SBE, only 11 (26%) exhibited a reduced SIDa. Similarly, among 61 patients with reduced SIDa, only 18% exhibited a reduced SBE. This can also be seen in Figure S2.

### 3.2. Admission vs. Day One

**Figure 1:**
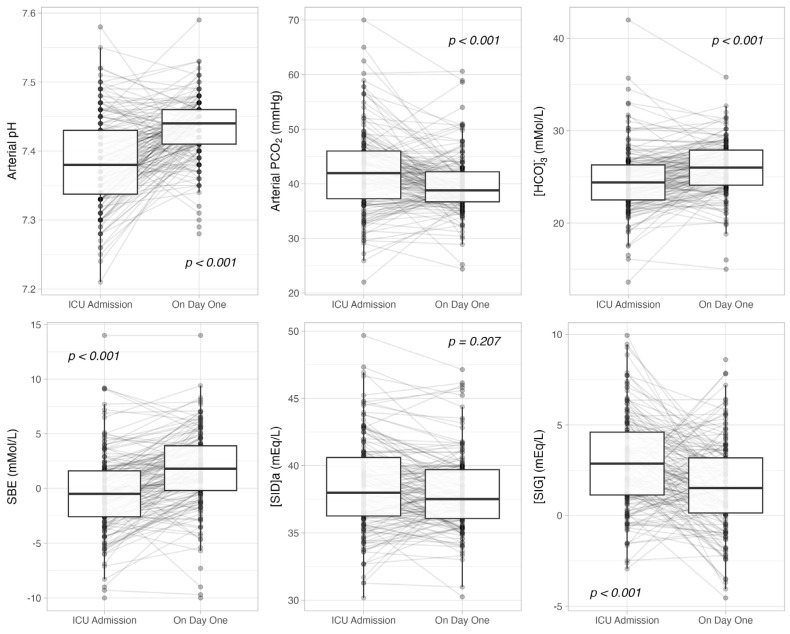
Time-course of arterial pH, carbon dioxide partial pressure (PCO_2_), bicarbonate concentration ([HCO_3_^−^]), standard base excess (SBE), apparent strong ion difference (SIDa) and strong ion gap ([SIG]) from ICU admission to day one.

Figure 1 shows the acid–base equilibrium according to the SBE and Stewart approaches on ICU admission and on day one. On day one, pH, HCO_3_ and SBE were significantly higher while PaCO_2_ was lower compared to the ICU admission. SIDa did not change while albumin and SIG significantly decreased. In terms of urinary acid–base, the urinary SID significantly increased with a concomitant increased retention of sodium and chloride with increased potassium excretion (Table S2). The changes from ICU admission to day one in terms of SIDa (ΔSIDa) were associated with the changes in SBE (ΔSBE) (*p* = 0.016) in a univariate model (R^2^ = 0.03, see Table S3); they remained independently associated with ΔSBE, changes in serum albumin (ΔAlbumin) and changes in PaCO_2_ (ΔPaCO_2_) from ICU admission to day one in a multivariate model (see Table S3); however, ΔSBE, even if adjusted for the other covariates, explained only a small ΔSIDa variance (R^2^ = 0.16).

### 3.3. On Day One

According to the arterial pH, 12 (7%), 48 (28%) and 112 (65%) patients had acidemia, alkalemia and a normal pH, respectively, on day one. This can also be seen in Figure S2.

On day one, the occurrence of metabolic acidosis according to the SBE and Stewart approaches (13% vs. 36%, respectively) was statistically different (*p* = 0.034); similarly, the rate of metabolic alkalosis was different (35% vs. 16%, respectively) (Table 5). The agreement between methods in detecting the concordance of acid–base derangement classes was estimated as poor (k = 0.07); indeed, among 20 patients with reduced SBE, only 9 (45%) exhibited a reduced SIDa. Among 61 patients with reduced SIDa, only 15% exhibited a reduced SBE. Comparing continuous values of SBE and Δnormal–actualSIDa, a bias of 3.9 with limits of agreement of −4.7 and 12.6 mEq/L were found, indicating poor agreement between the two values (Figure S1); accordingly, LCCC showed a poor concordance (0.01), due to the presence of outliers.

### 3.4. Patients with and Without Renal Impairment

On ICU admission, 91 patients (52%) had an altered glomerular filtration rate (eGRF). Patients with low eGRF showed a lower SBE and higher SIG compared to patients with normal renal function; accordingly, the pH was slightly lower (Table S7). On day one, the two groups presented a similar difference in BE and SIG. Urinary output was significantly lower in patients with low eGRF on ICU admission and on day one.

### 3.5. Diagnostic Accuracy of SBE and Apparent SID on Metabolic Acid–Base Derangements

Model performance, sensitivity and specificity, as well as the best threshold according to the Youden index and AUC of univariate logistic regression between categories of pH and SBE or SIDa are presented in Table S5. As shown, the comparison between AUC reveals a better predictive power of SBE as compared to SIDa. Similarly, model performance, sensitivity and specificity, as well as the best threshold according to the Youden index and AUC of the two multivariate models evaluating SBE or apparent SIDa, adjusting for relevant covariates (albumin, PaCO_2_, amount of administered fluids, creatinine), are presented in Tables S6 and S7, respectively. The comparison between models in terms of AUC reveals that the adjusted model based on SBE is an excellent predictor of acidemia or alkalemia, better than the apparent SID-based model.

## 4. Discussion

The main findings of the present study, which evaluated a mixed population of critically ill patients, are as follows: (1) on ICU admission 32% and 17% patients showed acidemia and alkalemia, while after 24 h, 7% and 28% patients had acidemia and alkalemia; (2) on ICU admission the occurrence of metabolic acidosis and metabolic alkalosis according to the SBE and Stewart approaches was similar (24% vs. 35%; 16% vs. 23%), with poor agreement between methods as well as on day one, (3) on ICU admission, acidemic patients had higher PaCO_2_ and lower SBE with similar SIDa but higher SIG, while alkalemic patients had lower plasma albumin; (4) on day one, in the overall population pH, HCO_3_ and SBE were increased while PaCO_2,_ albumin and SIG decreased with similar SIDa; and (5) patients with a reduction in the glomerural filtration rate both on ICU admission and day one had a significantly lower pH, SBE and SIG compared to patients with normal renal function.

In normal conditions, the arterial blood pH ranges between 7.36 and 7.44 and it is strictly regulated by the lung and the kidney system, in order to maintain the normal homeostasis and cellular function. Critically ill patients might have several disorders of the acid–base equilibrium significantly affecting the pH, with an associated high mortality due to underlying potentially life-threatening conditions [1,25]. Furthermore, a pH in the normal range does not exclude multiple acid–base disorders. Thus, in daily critical practice, a whole acid–base evaluation is necessary for a correct diagnosis and patient management.

Nowadays, there is some controversy about the most adequate approach to investigate the measurement of the metabolic component of acid–base derangements.

For more than 60 years, physicians have used the traditional approach based on the Henderson–Hasselbach equation, which assumes that the pH is related to the concentration of HCO_3_ and PCO_2_ [10]. According to this approach, all the causes of acidemia are due to an increase in PCO_2_ (respiratory acidosis) or to a reduction in HCO_3_ (metabolic acidosis) and the opposite for the alkalemia. Thus, the possible acid–base disorders are divided into four categories: metabolic acidosis, metabolic alkalosis, respiratory acidosis and respiratory alkalosis [1]. Subsequently, in the early 1980s, Stewart introduced the physico-chemical quantitative approach, in which the pH is regulated by three independent variables: the PCO_2_, the strong ion difference (i.e., SIDa) and the weak non-volatile acids [15,26]. The weak non-volatile acids are mainly constituted of plasma protein and phosphates [18]. The SIDa is computed as the sum of the concentration of the strong cations (sodium, potassium, calcium, magnesium) minus the sum of strong anions (chloride and anions of organic acid). The normal value for the SIDa ranges between 40 and 42 meq/L [27]. According to the electroneutrality state, the positive charge should be compensated by an equal negative force, which corresponds to the weak acids (Atot). As the SIDa decreases or the total concentration of weak acids increases, the hydrogen concentration increases and pH decreases. The Stewart approach recognizes six different acid–base alterations: a low SIDa acidosis, high PCO_2_ acidosis, high total weak acids acidosis, high SIDa alkalosis, low PCO_2_ alkalosis and low total weak acids alkalosis [26].

In the present study, on ICU admission, the median pH was 7.38 [7.33–7.43]; patients with acidemia had a median pH 7.31 [7.28–7.33], due to high PCO_2_ acidosis and high total weak acids in terms of higher values of PCO_2_ 47 [44–51], higher plasma albumin level 3.5 [3.0–3.7] and also higher SIG 5.4 [3.9–7.1], compared to patients with alkalemia with median pH 7.48 [7.47–7.50].

Analyzing the type of acid–base disorders, the most frequent are the metabolic acidosis and metabolic alkalosis [27]. Previous data in critically ill patients showed that the two most common acid–base disorders were the metabolic acidosis and metabolic alkalosis, mainly related to the presence of hemodynamic instability, a hypoperfusion state, acute renal failure, the use of diuretics and a vigorous correction of shock using large quantities of crystalloids solution [2,4,25]. In addition, Souza et al. described the presence of respiratory alkalosis in up to 70% of COVID-19 related to a high minute ventilation [5]. Both of them were associated to an increased mortality [2,7,8].

Although in the present study, the rate of detection of metabolic acidosis and metabolic alkalosis was not different according to the Stewart and SBE approaches, on ICU admission, the rate of agreement was poor. A confounding factor is the presence of hypoalbuminemia, often reported in critically ill patients due to alteration in permeability, low hepatic production or urine loss, which can mask a significant amount of metabolic acidosis [28]. For example, when hyperchloremia and hypoalbuminemia are together present, if the reduction in negative charges due to hypoalbuminemia is equal to the increase in chloride, pH, HCO_3_ and SBE are within normal range. Therefore, these two pathologic processes would not be diagnosed if we used only the SBE method.

In uncomplicated patients, the traditional method can adequately detect the main acid–base disorder [16,17]. However, in critically ill patients, the presence of complex disorders makes this approach inadequate [29]. Boniatti et al., comparing the Stewart approach and the traditional method, found that the Stewart approach would add an additional diagnosis of acid–base disorders in 33% of the patients [30]. Furthermore, recent additional correction factors have been proposed based on the Stewart approach to improve the diagnostic accuracy [31].

Not only the presence of acid–base disorders at admission but also the evolution during the intensive care stay are significantly related to the outcome. Previous studies analyzing acid–base behavior during the first days on intensive care stay showed that survivors had a better pH, BE and SIDa compared to non-survivors [8,32]. However, in the present study, it was not possible to analyze survivors compared to not survivors due to the low mortality rate and small sample size; thus, the whole population was analyzed.

According to Stewart’s approach, the increase in pH from admission to day one was due to concomitant factors such as a significant decrease in albumin and PCO_2_ and a decrease in SIG, without any change in sodium and chloride. Two independent variables of pH, such as PCO_2_ and weak acids, lead to an increase in pH; moreover, the decrease in unmeasured anions provides an increase in pH. The source of these unmeasured anions is unclear. It has been shown that high levels of SIG at ICU admission can be due to the presence of sepsis, as well as renal and hepatic dysfunction, and are also probably a marker of tissue hypoperfusion [33]. In our population, we can explain the pH shift from acidosis to alkalosis from ICU admission to day one by two contemporary mechanisms. Regarding the reduction in PaCO_2_, patients admitted for post-operative care increased their minute ventilation on day one due to reduced sedation and the associated shift from controlled to assisted ventilation with an increase in minute ventilation (7.1 [6.0–8.2] vs. 7.8 [6.5–9.3] L/min, *p* < 0.001), whereas we can hypothesize a reduction in CO_2_ production after 24 h of sedation in patients requiring care for acute medical or surgical conditions. With regard to metabolic components, patients received 1510 ± 740 mL of balanced crystalloid solutions from ICU admission to day one, almost equally divided between Ringer’s lactate and Rehydrating III; this could explain the alkalinizing effect associated with the decrease in albumin and unmeasured anions due to the hemodilution effect, secondary to fluid resuscitation.

According to the traditional approach, the same increase in pH from admission to day one was secondary to an increase in PCO_2_, HCO_3_ and SBE. Similarly, we can conclude that both respiratory and metabolic components are responsible of the pH variations; however, no information can be provided on the underlying mechanisms. In fact, SBE is a composite marker, derived from several contributing components (e.g., chloride, lactate, albumin, ketoacids) that could co-operate in opposite directions, dumping eachother’s effect.

As previously demonstrated, Mahlue et al. found that the increases in pH and BE from admission up to 21 days were mainly due to an increase in bicarbonate without any change in plasma sodium [34]. Lindner et al. showed in a small population of critically ill patients that peak plasma sodium was recorded in all patients after 3 days, the last day with normal serum sodium [3]. The development of hypernatremia was associated to metabolic alkalosis, while the other determinants of acid–base remained unchanged.

In our study, the association between changes in SID and changes in SBE from ICU admission to day one in the univariate model was poor; even if we performed a multivariate model adjusting for covariates, the variance explained remained low. Although BE is a composite marker that may be affected by changes in albumin, electrolyte concentration and fixed acids, which may cooperate in opposite directions to determine pH derangements, our results show it to be an excellent predictor of acidemia or alkalemia in patients without primary respiratory disorders, both independently and when adjusted for potential confounders. Similarly, apparent SID, per se, reflecting only a fraction of the causes of metabolic acidosis explained by alterations in BE, exhibited poor diagnostic accuracy for metabolic derangement; however, when adjusted for potential confounders, the diagnostic performance of apparent SID improved markedly. As Stewart has already shown, the apparent SID helps us identify the cause of metabolic acidosis, but it can only explain part of the multiple causes of metabolic acidosis.

In order to better understand acid–base behavior in the different clinical contexts of critically ill patients, we also analyzed the population according to the presence of a renal impairment, defined as an estimated GFR less than 80 mL/min. Typically, these patients, in addition to a lower amount of urine production, are characterized by metabolic acidosis, which is multifactorial and complex [35]. Metabolic acidosis results from the balance among the acidifying effect of unmeasured anions as well as hyperphosphatemia, hyperlactatemia, hypocalcemia and alkalinizing forces of hypoalbuminemia, hyperkalemia [1,36]. Previous data showed that unmeasured anions can be associated in 50–60% of patients with metabolic acidosis [37]. To better evaluate the acid–base disorders in patients with renal failure, the computation of the SIDe has been suggested, because it takes into account the role of weak acids (CO_2_, albumin and phosphate) and allows the computation of SIG. In healthy subjects, the SIDa and SIDe are equal, while in critically ill patients, they can be significantly different [27]. In the present study, patients with renal impairment had a significantly lower pH accompanied with lower SBE, lower SIDe and higher SIG, which persisted on day one compared to patients with normal renal function.

## 5. Limitations

This study has several limitations: (1) the lack of standardized treatment protocols before ICU admission in terms of fluid administration and vasoactive drugs use; (2) the absence of renal function data prior to ICU admission in acutely ill patients, which may have contributed to overestimating the impairment of kidney function; (3) the small number of covariates recorded during the study period.

## 6. Conclusions

The present study showed that in a mixed population of mechanically ventilated patients, the acid–base derangements were present in up to fifty percent, mainly due acidemia on ICU admission and to alkalemia after 24 h. Acidemia was mainly due to higher PaCO_2_, lower SBE, with similar apparent SID; alkalemia was mainly due to a decrease in PaCO_2_ albumin and SIG on day one. Moreover, after 24 h, the urinary SID significantly increased with a concomitant increased retention of sodium and chloride with increased potassium excretion. The presence of renal failure significantly contributed to acidemia both on ICU admission and after 24 h.

According to the present data, the agreement between the SBE and Stewart’s apparent SID in detecting metabolic disturbances was poor, probably because SBE is a composite marker, derived from several components and apparent SID constitutes only a part of the possible causes of acidosis or alkalosis detected by SBE. However, concerning the changes in acid–base equilibrium, the shift towards alkalemia on day one could be explained by the Stewart approach, in terms of decrease in albumin and unmeasured anions due to the hemodilution effect, secondary to fluid resuscitation. In fact, the Stewart approach could be more useful in detecting the causes of the variations over time of acid–base disturbances, especially in critically ill patients characterized by changes of mechanical ventilation and fluid administration.

Further perspectives could be derived from the application of the Stewart approach in a controlled experimental setting, where fluid administration and urine analysis could be strictly monitored.

## Figures and Tables

**Table 1:** Demographic, anthropometric and clinical data in the study population. SOFA: sequential organ failure assessment; APACHE: acute physiologic assessment and chronic health evaluation. Continuous data are reported as mean ± SD or median [IQR]; categorical data are reported as % (n).

	n = 172
Age, years	69 [57–77]
Female sex, % (n)	34.9 (60)
Weight, kg	75 [63–88]
Body mass index, kg/m^2^	26 [23–29]
SOFA score	3 [1–6]
APACHE III score	9 [7–13]
Reason for ICU admission, % (n)	
Acute respiratory failure	16.9 (29)
Sepsis	12.8 (22)
Post-operative	70.3 (121)
History of, % (n)	
Hypertension	51.7 (89)
Chronic kidney disease	4.7 (8)
Diabetes mellitus	6.4 (11)
Liver disease	16.3 (28)
Place prior to ICU admission, % (n)	
Emergency department	19.2 (33)
Operating room	70.3 (121)
Ward	10.5 (18)
Diuretic home therapy, % (n)	6.4 (11)
Hospital mortality, % (n)	14.0 (24)

**Table 2:** Plasmatic acid–base variables at ICU admission. PaCO_2_: arterial carbon dioxide partial pressure; [HCO_3_^−^]: arterial bicarbonate concentration; SID: strong ion difference. Data are reported as mean ± SD or median [IQR].

	n = 172
Arterial pH	7.38 [7.33–7.43]
PaCO_2_, mmHg	42 [37–46]
[HCO_3_^−^], mMol/L	24.5 [22.6–26.6]
Standard Base Excess, mMol/L	−0.6 [−3.0–1.7]
Apparent SID, mEq/L	39.2 [37.5–41.8]
Effective SID, mEq/L	35.7 [33.3–38.0]
SIG, mEq/L	3.7 [1.8–5.7]
Lactate, mMol/L	1.6 [1.2–2.4]
Albumin, g/dL	3.2 [2.8–3.7]
Phosphate, mg/dL	3.8 [3.2–4.4]
Sodium, mEq/L	137 [135–139]
Potassium, mEq/L	4.1 [3.8–4.5]
Calcium, mEq/L	1.17 [1.14–1.20]
Magnesium, mEq/L	2.2 [2.0–2.5]
Chloride, mEq/L	104 [102–106]

**Table 3:** Acid–base variables according to acidemia (pH < 7.35), alkalemia (pH > 7.45) or normal pH. PaCO_2_: arterial carbon dioxide partial pressure; [HCO_3_^−^]: arterial bicarbonate concentration; SID: strong ion difference. Data are reported as mean ± SD or median [IQR]. *: *p* < 0.050 vs. pH < 7.35; °: *p* < 0.050 vs. 7.35 ≤ pH ≤ 7.45. Bold: *p* < 0.001.

	pH < 7.35 32% (55)	7.35 ≤ pH ≤ 7.45 51% (88)	pH > 7.45 17% (29)	*p*
***Plasmatic***				
Arterial pH	7.31 [7.28–7.33]	7.39 [7.37–7.42] *	7.48 [7.47–7.50] *°	**<0.001**
PaCO_2_, mmHg	47 [44–51]	41 [38–44] *	35 [33–37] *°	**<0.001**
[HCO_3_^−^], mMol/L	23.7 [21.4–25.5]	24.6 [22.8–26.5] *	26.3 [23.8–28.0] *	**<0.001**
Standard Base Excess, mMol/L	−3.0 [−5.1–−0.8]	−0.4 [−2.2–1.7] *	3.5 [ 0.1–4.5] *°	**<0.001**
Apparent SID, mEq/L	40.4 [38.2–42.2]	38.8 [37.2–41.1]	38.5 [37.1–41.7]	0.112
Effective SID, mEq/L	34.6 [32.2–37.5]	35.6 [33.6–38.2]	37.5 [33.3–39.7] *	**0.024**
SIG, mEq/L	5.4 [3.9–7.1]	3.2 [1.4–4.6] *	2.5 [1.2–5.0] *	**<0.001**
Lactate, mMol/L	1.7 [1.2–2.4]	1.6 [1.1–2.4]	1.3 [1.0–1.7]	0.086
Albumin, g/dL	3.5 [3.0–3.7]	3.2 [2.8–3.7]	3.1 [2.7–3.4] *	**0.021**
Phosphate, mg/dL	4.1 [3.6–4.7]	3.8 [3.2–4.4]	3.4 [2.6–4.0] *	**0.002**
Sodium, mEq/L	138 [136–140]	137 [135–138] *	136 [134–141]	**0.033**
Potassium, mEq/L	4.2 [4.0–4.7]	4.2 [3.9–4.5]	3.7 [3.5–4.0] *°	**<0.001**
Calcium, mEq/L	2.4 [2.3–2.4]	2.3 [2.3–2.4] *	2.3 [2.2–2.4] *	**<0.001**
Magnesium, mEq/L	1.8 [1.6–2.0]	1.8 [1.6–2.1]	1.7 [1.6–1.9]	0.222
Chloride, mEq/L	104 [102–106]	104 [102–106]	104 [101–106]	0.867
**Urinary**				
Urine output, mL/kg/h	0.6 [0.3–1.3]	0.9 [0.5–1.6]	0.8 [0.4–1.0]	0.343
Urinary pH	6.5 [6.0–6.5]	6.5 [6.5–6.5]	6.5 [6.5–6.5]	0.519
Urinary SID, mEq/L	56 [35–72]	57 [36–77]	59 [38–103]	0.167
Urinary sodium, mEq/L	106 [70–150]	128 [83–155]	95 [68–143]	0.131
Urinary potassium, mEq/L	44 [31–58]	38 [24–58]	40 [24–57]	0.660
Urinary chloride, mEq/L	94 [48–126]	105 [68–137]	63 [26–115] °	**0.009**

**Table 4:** Prevalence of acid–base disturbances according to standard base excess and strong ion difference on ICU admission. Data are reported as % (n).

	*On ICU Admission*			
κ = 0.08		Standard Base Excess		
		Low	Normal	High
Strong Ion Difference	Low	11 (19)	22 (38)	2 (3)
	Normal	10 (18)	26 (44)	6 (11)
	High	3 (5)	12 (21)	8 (13)

**Table 5:** Prevalence of acid–base disturbances according to standard base excess and strong ion difference on day one. Data are reported as % (n).

	*On Day One*			
κ = 0.07		Standard Base Excess		
		Low	Normal	High
Strong Ion Difference	Low	5 (9)	22 (39)	8 (13)
	Normal	6 (10)	28 (47)	17 (29)
	High	1 (2)	3 (6)	10 (18)

## Data Availability

The dataset used in this study is available upon reasonable request to the corresponding author.

## Supplementary Materials

The following supporting information can be downloaded at: https://www.mdpi.com/article/10.3390/jcm14093227/s1. Table S1: Urinary acid-base variables at ICU admission; Table S2: Plasmatic acid-base variables at ICU admission and after 24 h; Table S3: Univariate and multivariate linear models to predict changes in strong ion difference (ΔSIDa); Table S4: Behavior of acid-base variables from admission to after 24 h according to renal function at admission; Table S5: Univariate logistic models to investigate strong ion difference (ΔSIDa) and SBE performance in detecting acidemia (pH < 7.36) or alkalemia (pH > 7.44) in patients without primary respiratory acid-base disorder; Table S6: Multivariate logistic models to investigate SBE performance in detecting acidemia (pH < 7.36) or alkalemia (pH > 7.44) in patients without primary respiratory acid-base disorder adjusted for covariates; Table S7: Multivariate logistic models to investigate apparent SID performance in detecting acidemia (pH < 7.36) or alkalemia (pH > 7.44) in patients without primary respiratory acid-base disorder adjusted for covariates. Figure S1: Acid-base characterisation flow chart; Figure S2: Prevalence of simple and mixed acid-base distubance according to SBE-based method (A) and according to Stewart’s metabolic derangement determinants (B); Figure S3: Bland-Altman plots comparing SBE and Δnormal-actualSIDa (the difference between a normal SIDa assumed as 42 mEq/L and the actual SIDa).
