# Supplementary material for: Acid–Base Status in Critically Ill Patients: Physicochemical vs. Traditional Approach
Source: J Clin Med. 2025 May 6;14(9):3227. doi: 10.3390/jcm14093227 (PMC12072961; doi:10.3390/jcm14093227)
Supplement: Supplementary file 1 [file jcm-14-03227-s001.zip › jcm-3547763-supplementary.pdf]

Journal of Clinical Medicine

MDPI

Supplementary Digital Material

Acid-Base Status in Criticall Ill Patients: Physicochemical *vs* Traditional Approaches

Arianna Ciabattoni [1], Davide Chiumello [1,2], Simone Mancusi [2], Tommaso Pozzi [2], Alessandro Monte [2], Cosmo Rocco [2] and Silvia Coppola [1,*]

[1] Department of Anesthesia and Intensive Care, ASST Santi Paolo e Carlo, San Paolo University Hospital, 20142 Milan, Italy; arianna.ciabattoni@asst-santipaolocarlo.it (A.C.); davide.chiumello@unimi.it (D.C.)
[2] Department of Health Sciences, University of Milan, 20122 Milan, Italy; simone.mancusi@unimi.it (S.M.); tommaso.pozzi@unimi.it (T.P.); alessandro.monte@unimi.it (A.M.); cosmo.rocco@unimi.it (C.R.)
* Correspondence: silvia_coppola@libero.it

Table S1. Urinary acid-base variables at ICU admission. $PaCO_2$: arterial carbon dioxide partial pressure; $[HCO_3^-]$: arterial bicarbonate concentration; SID: strong ion difference. Data are reported as mean ± SD or median [IQR].

	n = 172
Urine output, *mL/kg/h*	0.7 [0.4 – 1.4]
Urinary pH	6.5 [6.0 – 6.5]
Urinary SID, *mEq/L*	58 [36 – 78]
Urinary sodium, *mEq/L*	115 [75 – 153]
Urinary potassium, *mEq/L*	41 [26 – 58]
Urinary chloride, *mEq/L*	98 [56 – 132]

Table S2. Plasmatic acid-base variables at ICU admission and after 24 hours. $PaCO_2$: arterial carbon dioxide partial pressure; $[HCO_3^-]$: arterial bicarbonate concentration; SID: strong ion difference. Data are reported as mean ± SD or median [IQR].

	ICU admission	After 24 hours	*p*
Arterial pH	7.38 [7.33 – 7.43]	7.43 [7.40 – 7.46]	***<0.001***
$PaCO_2$, *mmHg*	42 [37 – 46]	39 [37 – 42]	***<0.001***
$[HCO_3^-]$, *mMol/L*	24.5 [22.6 – 26.6]	26.0 [24.1 – 28.0]	***<0.001***
Standard Base Excess, *mMol/L*	-0.6 [-3.0 – 1.7]	1.6 [-0.5 – 4.2]	***<0.001***
Apparent SID, *mEq/L*	39.2 [37.5 – 41.8]	39.0 [37.3 – 41.0]	*0.207*
Effective SID, *mEq/L*	35.7 [33.3 – 38.0]	36.7 [34.2 – 39.3]	***<0.001***
SIG, *mEq/L*	3.7 [1.8 – 5.7]	2.5 [0.9 – 4.6]	***<0.001***
Lactate, *mMol/L*	1.6 [1.2 – 2.4]	1.3 [1.1 – 1.9]	***0.003***
Albumin, *g/dL*	3.2 [2.8 – 3.7]	3.0 [2.7 – 3.3]	***<0.001***
Phosphate, *mg/dL*	3.8 [3.2 – 4.4]	3.8 [3.3 – 4.4]	*0.303*
Sodium, *mEq/L*	137 [135 – 139]	136 [134 – 138]	***<0.001***
Potassium, *mEq/L*	4.1 [3.8 – 4.5]	4.1 [3.9 – 4.4]	*0.729*
Calcium, *mEq/L*	1.17 [1.14 – 1.20]	1.15 [1.12 – 1.18]	***<0.001***

Magnesium, *mEq/L*	2.2 [2.0 – 2.5]	2.1 [1.9 – 2.3]	**<0.001**
Chloride, *mEq/L*	104 [102 – 106]	104 [102 – 106]	*0.154*
Urinary			
Urine output, *mL/kg/h*	0.7 [0.4 – 1.4]	0.6 [0.4 – 0.9]	**0.007**
Urinary pH	6.5 [6.0 – 6.5]	6.5 [6.0 – 6.5]	*0.614*
Urinary SID, *mEq/L*	58 [36 – 78]	60 [42 – 88]	**0.026**
Urinary sodium, *mEq/L*	115 [75 – 153]	97 [51 – 141]	**<0.001**
Urinary potassium, *mEq/L*	41 [26 – 58]	51 [36 – 72]	**<0.001**
Urinary chloride, *mEq/L*	98 [56 – 132]	92 [45 – 124]	**0.025**

Table S3: Univariate and multivariate linear models to predict changes in strong ion difference (ΔSIDa). β: linear coefficient; OR: odds ratio; CI: confidence intervals; ΔSBE: changes in SBE from ICU admission to day one; ΔAlbumin: changes in plasma albumin from ICU admission to day one; $\Delta PaCO_2$: changes in $PaCO_2$ from ICU admission to day one; ΔCreatinine: changes in plasmatic creatinine from ICU admission to day one.

**Univariate model (R^2 = 0.03)**			
**Variable**	**β**	***p***	**OR (95% CI)**
ΔSBE	0.16	0.016	1.17 (1.03 – 1.34)
**Multivariate Model (R^2 = 0.16)**			
ΔSBE	0.19	0.003	1.21 (1.07 – 1.38)
ΔAlbumin	1.77	<0.001	5.87 (2.15 – 16.05)
$\Delta PaCO_2$	0.10	<0.001	1.11 (1.05 – 1.17)
Administered Fluids	$-7.64\ 10^{-5}$	0.789	0.99 (0.99 – 1.01)
ΔCreatinine	0.16	0.500	1.17 (0.74 – 1.86)

Table S4. Behavior of acid-base variables from admission to after 24 hours according to renal function at admission. $PaCO_2$: arterial carbon dioxide partial pressure; $[HCO_3^-]$: arterial bicarbonate concentration; SIDa: strong ion difference. Data are reported as mean ± SD or median [IQR].

	**Normal eGFR 48% (84)**	**Altered eGFR 52% (91)**	p_{GR}	p_{TIME}	p_{INT}
Plasmatic					
Plasma pH<br>Admission<br>Day 1	 7.39 [7.34 – 7.44]<br>7.43 [7.41 – 7.46]	 7.36 [7.33 – 7.42]<br>7.44 [7.38 – 7.46]	*0.062*	**<0.001**	*0.209*
$PaCO_2$, *mmHg*<br>Admission<br>Day 1	 43 [37 – 47]<br>39 [37 – 43]	 41 [38 – 45]<br>38 [36 – 42]	*0.086*	**<0.001**	*0.744*
$[HCO_3^-]$, *mMol/L*			**<0.001**	**<0.001**	*0.474*

Admission<br>Day 1	23.8 [21.6 – 25.7]<br>25.4 [22.3 – 27.3]	25.5 [23.6 – 27.0]<br>26.4 [24.9 – 28.4]			
Standard Base Excess, *mMol/L*<br>Admission<br>Day 1	0.2 [-1.5 – 2.3]<br>2.1 [0.3 – 4.4]	-1.9 [-3.9 – 0.5]<br>1.4 [-2.2 – 3.3]	***<0.001***	***<0.001***	*0.310*
Apparent SID, *mEq/L*<br>Admission<br>Day 1	39.7 [37.7 – 42.0]<br>39.0 [37.3 – 40.8]	39.0 [37.2 – 41.7]<br>38.9 [37.4 – 41.1]	*0.323*	*0.242*	*0.567*
Effective SID, *mEq/L*<br>Admission<br>Day 1	37.0 [34.5 – 39.0]<br>37.0 [35.2 – 39.7]	34.6 [32.2 – 37.2]<br>36.7 [33.3 – 38.2]	***<0.001***	***<0.001***	*0.099*
SIG, *mEq/L*<br>Admission<br>Day 1	2.9 [1.2 – 4.7]<br>1.9 [0.3 – 3.5]	4.4 [2.7 – 7.0]<br>3.3 [1.3 – 5.9]	***<0.001***	***<0.001***	*0.818*
Lactate, *mMol/L*<br>Admission<br>Day 1	1.6 [1.1 – 2.4]<br>1.3 [1.1 – 1.7]	1.6 [1.2 – 2.3]<br>1.4 [1.1 – 1.9]	*0.559*	***<0.001***	*0.540*
Albumin, *g/dL*<br>Admission<br>Day 1	3.3 [2.9 – 3.7]<br>3.0 [2.7 – 3.3]	3.2 [2.8 – 3.6]<br>3.0 [2.7 – 3.3]	*0.201*	***<0.001***	*0.196*
Phosphate, *mg/dL*<br>Admission<br>Day 1	3.8 [3.2 – 4.4]<br>3.8 [3.3 – 4.4]	3.9 [3.4 – 4.5]<br>3.8 [3.3 – 4.4]	*0.275*	*0.231*	*0.493*
Sodium, *mEq/L*<br>Admission<br>Day 1	137 [135 – 139]<br>136 [134 – 138]	137 [135 – 140]<br>136 [135 – 138]	*0.551*	***<0.001***	*0.877*
Potassium, *mEq/L*<br>Admission<br>Day 1	4.0 [3.7 – 4.4]<br>4.1 [3.8 – 4.4]	4.2 [3.9 – 4.6]<br>4.2 [4.0 – 4.6]	*0.002*	*0.640*	*0.621*
Calcium, *mEq/L*<br>Admission<br>Day 1	1.17 [1.14 – 1.20]<br>1.15 [1.13 – 1.18]	1.17 [1.14 – 1.20]<br>1.15 [1.11 – 1.19]	*0.707*	***<0.001***	*0.832*
Magnesium, *mEq/L*<br>Admission<br>Day 1	2.1 [2.0 – 2.4]<br>2.1 [1.9 – 2.3]	2.2 [2.0 – 2.5]<br>2.2 [1.9 – 2.4]	*0.336*	***<0.001***	*0.536*
Chloride, *mEq/L*<br>Admission<br>Day 1	104 [103 – 106]<br>103 [101 – 105]	104 [103 – 106]<br>104 [102 – 106]	***0.010***	***0.030***	*0.995*
Urinary					
Urine output, *mL/kg/h*<br>Admission<br>Day 1	0.9 [0.5 – 1.6]<br>0.7 [0.5 – 1.0]	0.6 [0.2 – 1.0]<br>0.5 [0.4 – 0.9]	***<0.001***	***0.042***	***0.002***
Urinary SID, *mEq/L*			*0.084*	***0.022***	*0.163*

Admission<br>Day 1	58 [38 – 81]<br>69 [48 – 90]	56 [36 – 71]<br>54 [36 – 77]			
Urinary sodium, *mEq/L*<br>Admission<br>Day 1	 127 [82 – 158]<br>98 [59 – 140]	 104 [65 – 144]<br>94 [41 – 141]	*0.112*	*0.002*	*0.439*
Urinary potassium, *mEq/L*<br>Admission<br>Day 1	 39 [24 – 58]<br>57 [41 – 75]	 44 [28 – 57]<br>46 [33 – 61]	*0.093*	***<0.001***	***<0.001***
Urinary chloride, *mEq/L*<br>Admission<br>Day 1	 107 [65 – 137]<br>97 [51 – 129]	 87 [53 – 123]<br>87 [44 – 121]	*0.068*	*0.109*	*0.974*

Table S5: Univariate logistic models to investigate strong ion difference (ΔSIDa) and SBE performance in detecting acidemia (pH < 7.36) or alkalemia (pH > 7.44) in patients without primary respiratory acid-base disorder. β: linear coefficient; OR: odds ratio; CI: confidence intervals; sens: sensitivity; spec: specificity; threshold: best threshold value according to Youden index; AUC: area under the curve.

**pH ~**	**β**	***p***	**OR (95% CI)**	**sens**	**spec**	**threshold**	**AUC**
SBE	0.26	***<0.001***	0.77 (0.72 – 0.83)	0.84	0.61	1.2	0.78
aSID	0.04	*0.131*	1.05 (0.99 – 1.11)	0.36	0.78	38.9	0.55

Table S6: Multivariate logistic models to investigate SBE performance in detecting acidemia (pH < 7.36) or alkalemia (pH > 7.44) in patients without primary respiratory acid-base disorder adjusted for covariates. β: linear coefficient; OR: odds ratio; CI: confidence intervals; sens: sensitivity; spec: specificity; threshold: best threshold value according to Youden index; AUC: area under the curve.

	**β**	***p***	**OR (95% CI)**	**sens**	**spec**	**AUC**
SBE	-0.98	***<0.001***	0.37 (0.26 – 0.49)	0.97	0.95	0.99
Albumin	0.99	*0.075*	2.71 (0.94 – 8.67)			
$PaCO_2$	0.72	***<0.001***	2.05 (1.67 – 2.67)			
Administered fluids	-0.01	*0.589*	0.99 (0.99 – 0.99)			
Creatinine	0.33	*0.320*	1.39 (0.70 – 2.78)			
PEEP	-0.36	*0.834*	0.96 (0.69 – 1.36)			
Respiratory rate	-0.02	*0.761*	0.98 (0.86 – 1.12)			

Table S7: Multivariate logistic models to investigate apparent SID performance in detecting acidemia (pH < 7.36) or alkalemia (pH > 7.44) in patients without primary respiratory acid-base disorder adjusted for covariates. β: linear

coefficient; OR: odds ratio; CI: confidence intervals; sens: sensitivity; spec: specificity; threshold: best threshold value according to Youden index; AUC: area under the curve.

	β	*p*	OR (95% CI)	sens	spec	AUC
Apparent SID	-0.29	***<0.001***	0.75 (0.66 – 0.84)	0.82	0.84	0.88
Albumin	1.37	***<0.001***	3.93 (1.90 – 8.51)			
$PaCO_2$	0.29	***<0.001***	1.34 (1.23 – 1.47)			
Administered fluids	0.01	***0.036***	1.00 (0.99 – 1.01)			
Creatinine	0.70	***0.002***	2.01 (1.34 – 3.23)			
PEEP	0.08	*0.434*	1.08 (0.89 – 1.32)			
Respiratory rate	0.01	*0.861*	1.01 (0.92 – 1.10)			

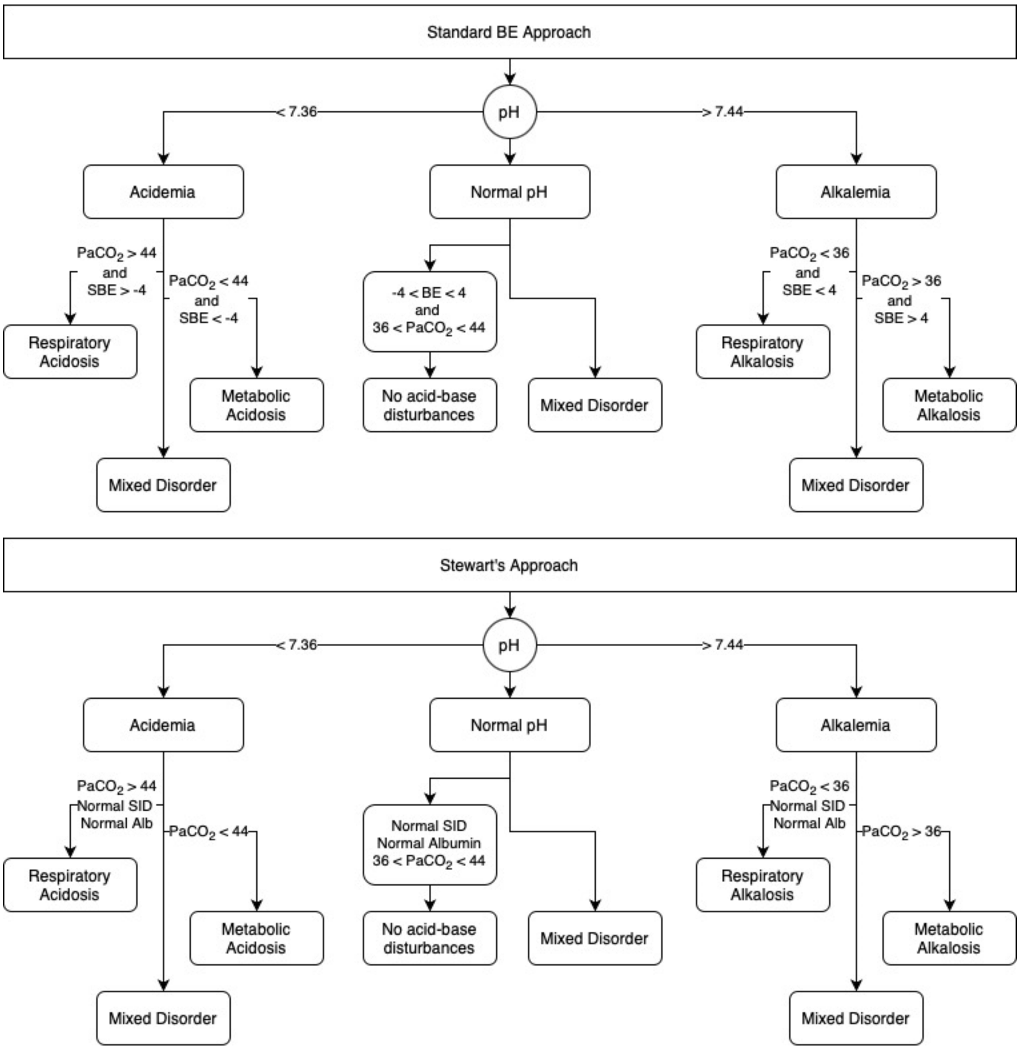

Figure S1. Acid-base characterisation flow chart.

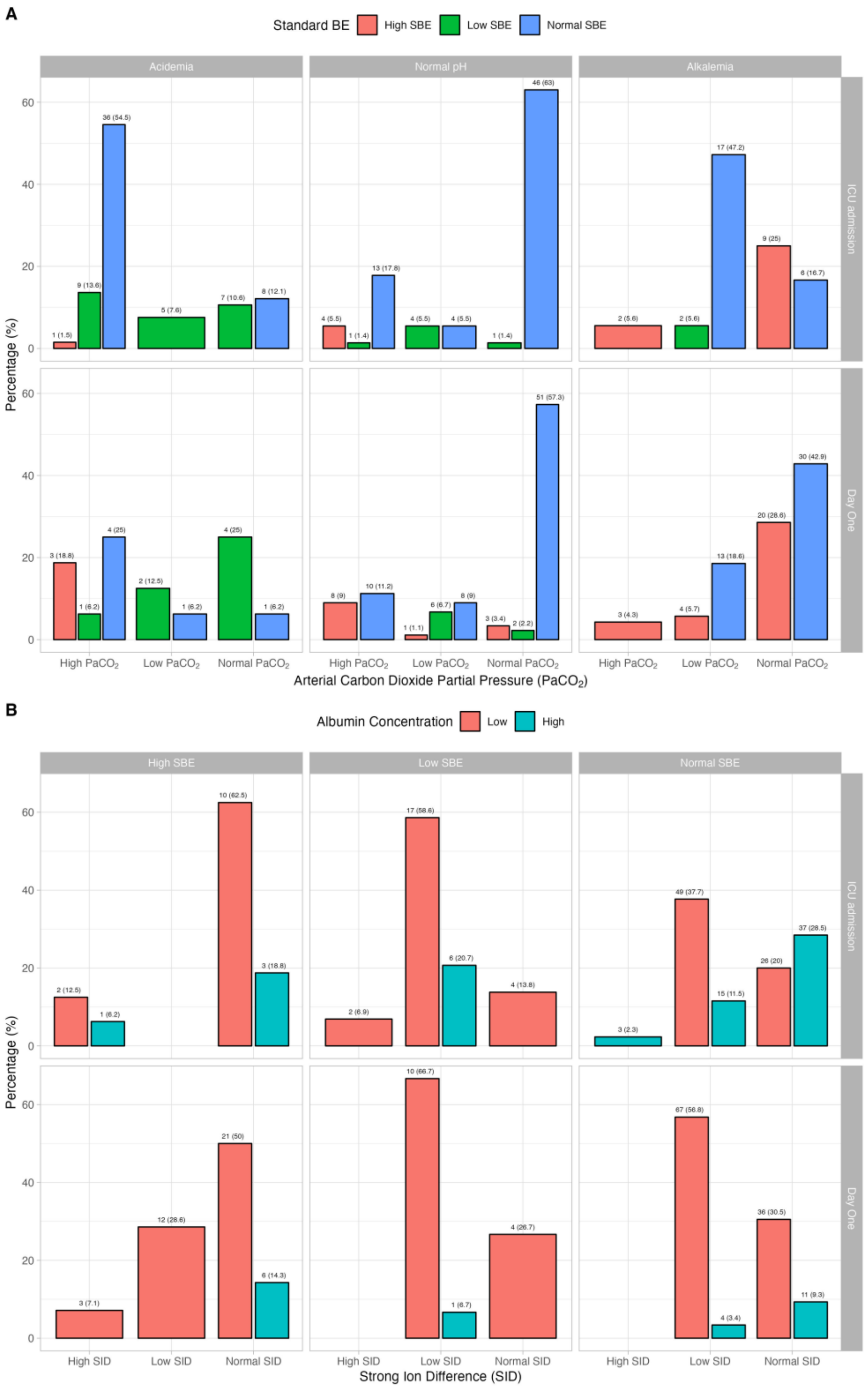

Figure S2. Prevalence of simple and mixed acid-base distubance according to SBE-based method (A) and according to Stewart's metabolic derangement determinants (B). Acidemia: pH < 7.36, normal pH: 7.36 – 7.44, alkalemia: pH > 7.44; high $PaCO_2$: > 44 mmHg; normal $PaCO_2$: 36 - 44 mmHg; low $PaCO_2$: < 36 mmHg; low SBE: < -4 mMol/L; normal SBE: -4 - 4 mMol/L; high SBE: > 4 mMol/L; low SIDa: < 38 mEq/L; normal SIDa: 38 - 42 mEq/L; high SIDa: > 42 mMol/L.

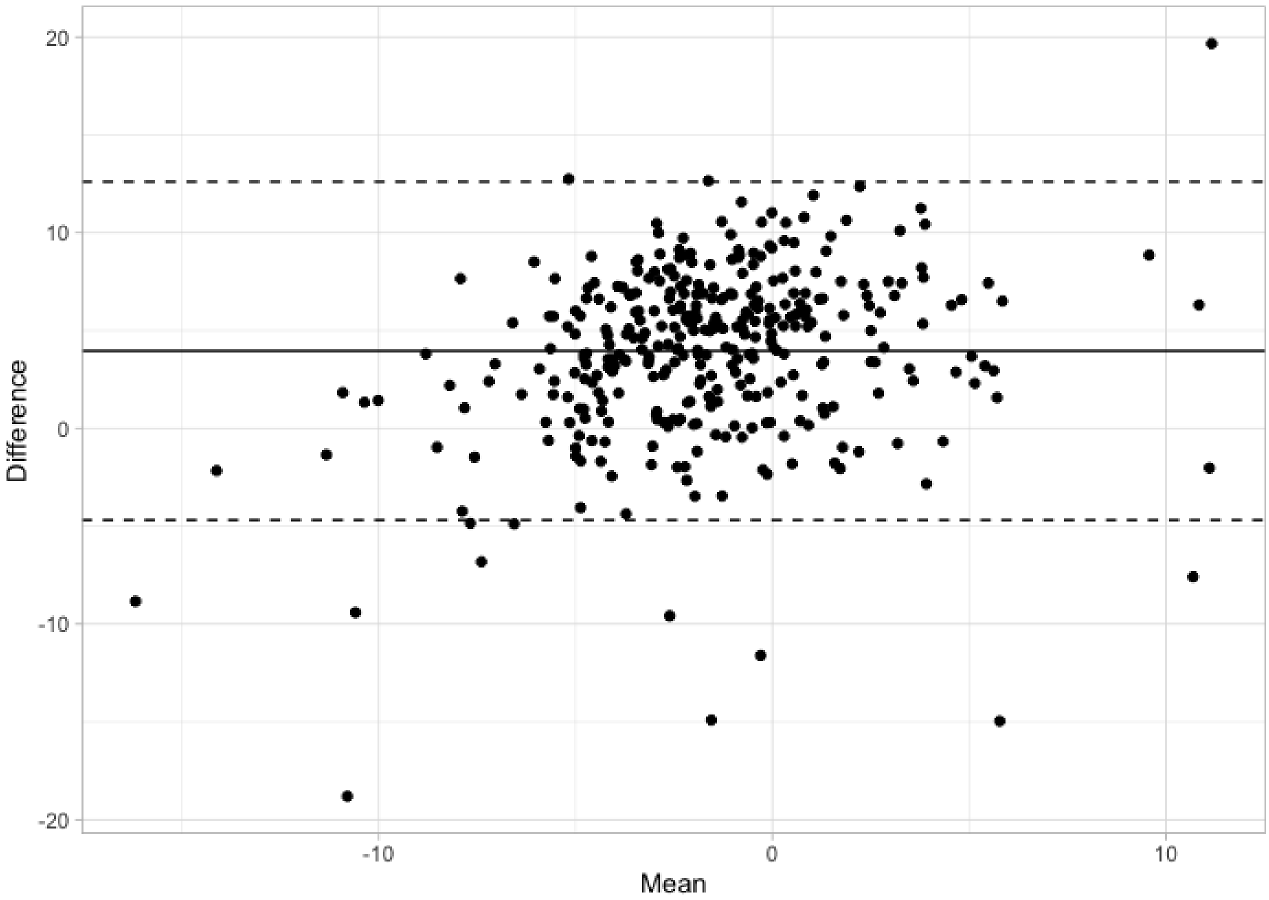

Figure S3. Bland-Altman plots comparing SBE and Δnormal-actualSIDa (the difference between a normal SIDa assumed as 42 mEq/L and the actual SIDa). Mean bias: 3.9, upper Limit of Agreement (LOA): 12.6, lower LOA: -4.7.
